# The Social Side of Sleep: A Systematic Review of the Longitudinal Associations between Peer Relationships and Sleep Quality

**DOI:** 10.3390/ijerph20032017

**Published:** 2023-01-21

**Authors:** Francesca De Lise, Valeria Bacaro, Elisabetta Crocetti

**Affiliations:** Department of Psychology “Renzo Canestrari”, Alma Mater Studiorum University of Bologna, 40126 Bologna, Italy

**Keywords:** adolescents, sleep quality, peer relationships, systematic review, longitudinal

## Abstract

In adolescence, peer relationships become crucial since youths start to rely on their peers for support. Thus, multiple facets of adolescents’ well-being are affected by their peer relationships. In this vein, one of the central well-being aspects that could be affected by the peer relationships of adolescents is sleep quality. Nevertheless, it is still unclear how multiple peer relationship factors (i.e., positive, negative, emotional, and behavioral issues related to peer relationships) are intertwined with adolescents’ sleep quality. For this reason, this systematic review with meta-analysis aims to summarize longitudinal studies to uncover how the interplay between peer relationship factors and adolescents’ sleep quality unfolds over time. Nineteen longitudinal studies involving a total of 21,232 adolescents were included. Overall, findings from this review showed that (a) positive peer relationships and sleep quality were not associated over time; (b) negative peer relationships and sleep quality were bidirectionally associated over time; (c) few studies evaluated the bidirectional relations between emotional and behavioral issues and sleep quality, showing links with sleep schedule and duration, but not with sleep quality. Meta-analytic results were discussed, considering their implications.

## 1. Introduction

Sleep is an essential part of everyone’s lives and reflects quite faithfully the state of well-being of an individual [1]. Notably, during adolescence, adequate sleep facilitates the regulation of critical daily functions such as behavior, emotion, and attention (for a review, [2]) which are a prerequisite for health and well-being [3]. At the same time, sleep represents a complex process determined by a wide range of biological, behavioral, social, and cultural factors, which can be examined from a developmental ecological systems perspective [4]. Individual characteristics, along with developmental tasks and demands of significant social contexts (e.g., family, school, peers), increase youth stress levels and could therefore interfere with their sleeping habits. In this vein, the peer context has an outstanding role since the quality of the relationships with peers strongly impacts several aspects of adolescents’ life [5]. 

Peer relationships are one of the primary sources of social support for adolescents. On the one hand, good peer relationships can improve social skills development, enhancing adolescents’ physical health and well-being [6]. On the other hand, negative peer relationships may have detrimental consequences on their academic performance, affecting their emotional well-being and mental health [7]. Thus, multiple facets of adolescents’ well-being are affected by their peer relationships [8].

One of the central well-being aspects that could be affected by the peer relationships of adolescents is sleep quality [9,10]. A growing literature has focused on the social side of sleep, highlighting the multiple associations and influences of different aspects of adolescents’ social life on sleep. Nevertheless, most research focused on the impact of negative (e.g., peer victimization; cybervictimization) and positive (e.g., social connections with peers; romantic relationships) relational aspects on sleep quality [11,12] while less attention has been paid to the reciprocal associations between peer relationships and sleep dimensions. Moving from these considerations, this systematic review with meta-analysis aims to summarize the longitudinal studies which explored the reciprocal associations between sleep quality and peer relationships in adolescents over time.

### 1.1. Peer Relationships in Adolescents

During adolescence, youths start to rely on their peers for support [13] and feel more comfortable confiding in them than their parents or other adults [14]. Adolescents interact with their peers in various contexts (i.e., school, sport, leisure activities, online exchanges), and these connections affect their lives, depending on the quality of the contact and how they perceive it. The experiences that adolescents have with their peers could be connotated by positive and negative characteristics affecting their developmental trajectories, highlighting protective (e.g., sports participation) and risk (e.g., peer victimization) factors for their health [15,16]. 

### 1.2. Positive Peer Relationships

Adolescents can experience positive connections with their peers, from relationships with classmates to daily connections with friends in leisure and sports time [17]. Moreover, during adolescence, individuals start to experience their first romantic relationships, which bring several life changes and start to have a significant impact on their well-being [18]. These interactions are essential to adolescents’ experiences and have a crucial role in their subjective perception of their quality of life. 

Generally, peer relationships can affect adolescents’ well-being differently based on the quality and quantity of the contact they enact. Adolescents experience the emotional and social roller coaster of changing groups of friends and building new bonds in the transition between middle and high school [19]. In this framework, social support from friends becomes increasingly crucial for social-emotional growth and adjustment [20] and a protective factor for adolescents’ mental health [14]. Furthermore, social relationships are an essential source of global self-esteem and vice versa [21]. The quality of romantic experience enhances self-esteem, self-confidence, and social competence (for a review, [22]). In contrast, poor-quality romantic relationships are detrimental to well-being outcomes [23].

### 1.3. Negative Peer Relationships

The other side of the coin of the changes that adolescents encounter in their social life is that they may struggle to feel the pressure to “fit in” within their peer context and that they might have negative experiences with their peers (e.g., peer victimization, cybervictimization). Negative peer relationships are related to the feeling of being rejected and can lead to various types of maladjustment. One of the most negative and frequent adolescent experiences is peer victimization, which is the repeated and systematic abuse of power by one or more peers over time in purposeful attempts to injure or inflict discomfort [24,25]. Youths victimized by their peers experience a heightened risk for adverse mental and physical health outcomes both concurrently and over time [26].

Furthermore, nowadays, social media and online connections are essential tools used by youth to stay in touch with their peers and create new relationships. If, on the one hand, social media can facilitate the creation of bonds among people, on the other hand, they can also expose users to the risk of adverse consequences in terms of cybervictimization and cyberbullying episodes (for a review, [27]). Cyberbullying has been defined as usually repeated aggressive and hostile messages intentionally sent through electronic media and is associated with a multitude of adverse problems similar to traditional bullying [28]. Specifically, victims of cyberbullying have been found to experience lower self-esteem, higher levels of depression, and experience significant life challenges [29]. 

### 1.4. Emotional and Behavioral Issues Related to Peer Relationships

Taking a step further, it is worth considering additional emotional and behavioral issues that are particularly important for understanding the quality of peer relationships. In this respect, two of the main crucial aspects are feelings of loneliness [30] and fear of missing out [31], which may impact the interactions they experience. Specifically, the subjective feeling of loneliness/being surrounded by others can differ from the objective quantity of friends [32]. Moreover, nowadays, adolescents are constantly connected through the powerful tool of social media, which provides a significant amount of information (e.g., activities, events, possibility of being in real-time contact with friends). This continuous flux of updates and contact with many diverse groups of people and many different stimuli can lead to a phenomenon known as fear of missing out. This dimension is defined as a pervasive apprehension that others might participate in enriching experiences from which one is absent, and it is characterized by the desire to stay continually connected with what others are doing [33]. As an indicator of unmet relatedness needs, fear of missing out may contribute to adverse health outcomes due to maladaptive self-regulatory behaviors for seeking gratification of such need [34]. 

Moreover, together with the onset of a romantic relationship, some adolescents start their first sexual experiences. For those who have their first sexual experiences during adolescence, lack of information and other factors such as depressive symptoms, use of substances, and family related issues can lead to risky sexual behaviors that might jeopardize adolescents’ health [35]. This issue may, in turn, lead to different kinds of maladjustment, increasing other risky activities as well as affecting the individuals’ general sense of well-being [36].

Overall, adolescents experience a broad pattern of interactions with their peers daily, representing one of the main parts of their everyday life. Thus, the positive and negative connotations of these relationships have a crucial impact on their general adjustment. Since sleep quality is an essential feature of one’s functioning, to better understand the mechanism underlying the impact of peer relationships on well-being and to reduce risk and enhance protective factors, it is necessary to look more deeply into the association of this socioecological level with sleep dimensions. 

### 1.5. The Reciprocal Influence between Peer Social Context and Sleep 

Good quality sleep is conceptualized as a multidimensional construct composed of subjective satisfaction with sleep, a regular sleep schedule, a proper amount of sleep duration, and ease of falling asleep and returning to sleep [37]. However, dramatic changes in sleep schedule, duration, and quality characterize adolescence as a consequence of biological variations (e.g., circadian sleep processes), unhealthy practices (e.g., evening screen time), and socio-contextual factors (e.g., school time) [38]. Thus, during adolescence, poor sleep quality and disturbances are widespread and are associated with substantial adverse physical and mental health outcomes [39,40,41]. 

Notably, the peer context could play a significant protective role in sleep quality. Most of the previous studies focused on the importance of social relationships on adults’ and emerging adults’ health, highlighting on the one hand that people with negative social ties report more sleep disturbances [42] and that, on the other hand, loneliness is associated with poorer sleep quality [43]. Recently, researchers have also turned their attention to the social side of sleep in adolescents, seeking to understand the links between sleep quality and peer relationships [44]. 

One of the most important factors is the changes that social relationships with peers bring into adolescents’ lives. For instance, being more popular in high school is associated with more insomnia symptoms, which might be related to the need to fulfill all friends’ expectations and the peer pressure of maintaining their popularity [45]. At the same time, psychosocial factors related to the peer system likely interact in nuanced and complex ways to influence youth’s sleep health over time [46], leading to cascading adverse outcomes. Moreover, it has been highlighted that social problems with peers, such as experiencing peer victimization, are associated with poorer physical health and more sleep problems [16].

Conversely, appropriate sleep quality and duration can impact adolescents’ relationships with their peers. For instance, adverse consequences of insufficient sleep, such as emotion-regulation deficits and irritability, may impact the quality of peer relationships [47]. Moreover, poor sleep quality could lead to emotional and behavioral difficulties, peer problems, and lower well-being which would, in turn, influence their social experience [48]. However, less attention has been paid to this direction, stressing the need to shed new light on this topic and provide a more nuanced picture of the interplay between sleep quality and peer relationships over time.

### 1.6. The Current Study

Moving from these considerations, it is of utmost importance to provide a comprehensive understanding of how different aspects of peer relationships might influence and be influenced by the multiple developmental aspects of sleep quality (i.e., adequate sleep duration, regular sleep schedule, sleep disturbances, and sleep quality). If, on the one hand, previous systematic reviews highlighted meaningful associations between peer relationships and adolescents’ sleep quality (e.g., [2,49]), on the other hand, they pointed to the need for more longitudinal evidence in order to understand how these associations unfold over time since a main limitation of the literature was the over-reliance on cross-sectional studies [2]. Thus, the current systematic review with meta-analysis aims to synthesize the longitudinal evidence on the interplay between peer relationships dimensions (i.e., positive, negative, and emotional and behavioral issues related to peer relationships) and sleep quality (i.e., sleep duration, sleep quality, sleep disturbances, sleep schedule) in adolescence.

## 2. Materials and Methods

This study was conducted following the PRISMA guidelines (Preferred Reporting Items for Systematic Reviews and Meta-Analyses; [50]). The PRISMA checklist is available in the Supplementary Materials (Document S1). This systematic review was preregistered in the PROSPERO database, registration ID: CRD42021281002. The current study is part of a larger project aiming to review longitudinal research investigating the interplay between sleep quality and several socio-contextual factors (e.g., family, school, macro-context) in adolescence.

### 2.1. Eligibility Criteria

Following the PRISMA guidelines [51], specific eligibility criteria were defined. With regards the study characteristics, studies were eligible for the systematic review if (a) participants were adolescents from the general population aged between 10/11 and 18/19 years old; (b) the study design was longitudinal (with at least two assessments, such as two-wave longitudinal studies or daily diaries); (c) the studies examined at least one aspect of peer relationship and one of sleep quality. Sleep could be measured with either objective (e.g., actigraphy, polysomnography) or subjective standardized measures (e.g., sleep diaries; questionnaires). Regarding the publication’s characteristics, journal articles and grey literature that can be retrieved through database searches (e.g., doctoral dissertations) were included to avoid selection biases and strengthen the methodological rigor of the systematic review [52]. Finally, no restrictions were applied based on the year and the language of publication (when articles/dissertations were published in a language other than English, professional translators were contacted).

### 2.2. Literature Search 

In order to systematically identify eligible relevant research published in peer-reviewed journal articles or available as grey literature, different search strategies were applied. First of all, several bibliographic databases were systematically searched until 23 September 2021: Web of Science, Scopus, PsycINFO, PsycArticles, PubMed, MEDLINE, ERIC, ProQuest Dissertations and Theses, and GreyNet. In each database, the following combination of keywords was searched for: (Sleep* OR insomnia* OR polysomnogra* OR REM OR actigraph* OR EEG* OR motor activity* OR circadian* OR chronotype*) AND (pediatr* OR paediatr* OR teen* OR school* OR adolescen* OR youth* OR young* OR child*) AND (longitudinal* OR prospective* OR follow up* OR daily* OR day-to-day OR wave*). Full query strings used in each database are reported in the Supplementary Material (Document S2).

This main bibliographic search was complemented with additional search strategies. The journals’ websites deemed most likely to publish studies on the topic were searched, identifying them using the statistics of the previous search on Web of Science. The fifteen journals in which most articles matching our search strategy had been published were identified (complete list of journals is provided in the Supplementary Materials Document S3) to screen in press articles (e.g., online first) that matched the eligibility criteria. Furthermore, conference proceedings from recent sleep-related journals were screened (*Journal of Sleep Research*, in which European Sleep Research Society Congress proceedings were published, and *Sleep Medicine*, in which the World Sleep Congress proceedings were published). The reference lists of the most relevant published systematic reviews and meta-analyses were checked (e.g., [53]; the complete list is given in the Supplementary Materials (Document S4). Finally, the reference lists of included studies were checked to further identify relevant studies not initially found through the other search strategies (this search was performed at the end of the selection process). The searches and the screening were run and managed on Citavi 6 software (V.6.14, Swiss Academic Software). 

### 2.3. Selection of Studies

**Figure 1:**
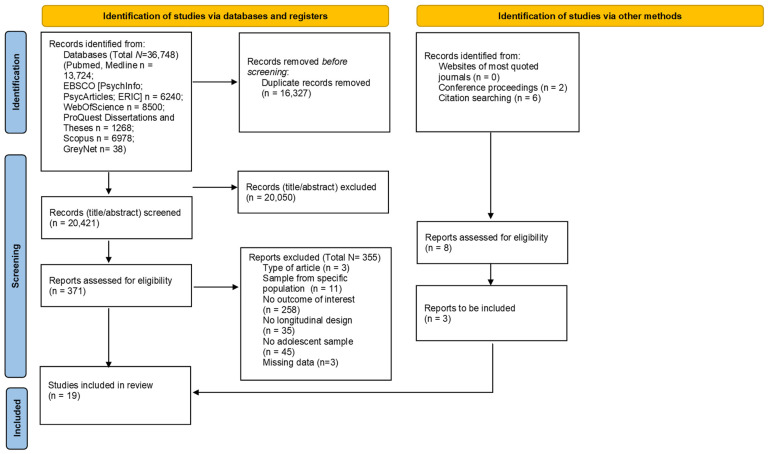
PRISMA diagram. From [54].

The results of the search strategies are reported in the PRISMA diagram (Figure 1). A total of 36,748 abstracts were identified, and 16,327 duplicates were removed. Two independent raters screened the remaining records (*n* = 20,421) independently and simultaneously. The percentage of agreement was substantial (Cohen’s Kappa = 0.81). Discrepancies were discussed with a third rater, and the final decisions were taken to reach an agreement among the three evaluators. 

A total of 371 records were selected at this step. Next, the full-texts were screened following the same procedure used for abstract screening (the agreement was high; Cohen’s Kappa = 0.61). In total, 19 studies were included in this systematic review.

### 2.4. Coding of Primary Studies

In order to extract relevant information from the selected primary studies, an excel spreadsheet was prepared. All the included studies were coded independently and simultaneously by two independent raters (the percentage of agreement was 95%). Discrepancies were discussed with a third rater and resolved among the three evaluators.

First, the characteristics of the publication were coded: type of publication (i.e., journal article or grey literature), year of publication, and the language of publication. Second, the characteristics of the studies were coded: funding sources (i.e., international funding, national funding, local funding, multiple funding sources); the number of waves of the longitudinal design; the interval between waves; the dimensions of each study; and the source of information used to evaluate them (i.e., self-reports, objective assessment). Third, the characteristics of the participants were coded: sample size, gender composition of the sample (% females), mean age, geographical location, and ethnic composition of the sample.

Finally, data necessary for effect size computations were extracted. Due to the high heterogeneity of the studies included, different effect sizes were coded (i.e., odds ratio, cross-lagged correlations, and beta coefficients) to address how peer relationships (i.e., positive and negative peer relationships, emotional and behavioral issues related to peer relationships) and sleep quality (sleep duration and schedule, sleep quality, and presence of sleep disturbances) were longitudinally related (see Strategy of analysis section). If only standardized beta regression coefficients were reported, the correlation coefficients were estimated based on Peterson and Brown’s formula [55]. When data for effect size computations were not reported in primary studies, the study authors were contacted by email to request missing data. In total, five authors were contacted to obtain all (or part of) the necessary data for effect size computations. If the authors did not answer the first request, three reminders (one every two weeks) were scheduled. Two authors replied, specifying that they could not provide the required data (e.g., they could no longer access the dataset), and three did not respond to the request. The total number of 19 studies included in the review accounts for three that were excluded because of insufficient data, as indicated in the PRISMA diagram (Figure 1). 

### 2.5. Strategy of Analysis

To address the research question, data related to peer relationships measured at one time point (e.g., positive peer relationships at T1) and sleep quality variables at a later time point (T2), or sleep quality variables at one time point (T1) and peer relationships variables at the following time point (e.g., cyberbullying at T2) were coded. When possible, the effect sizes were converted into Pearson’s correlations in order to compare the effects across studies and compute overall summary statistics through meta-analytic techniques. Pearson’s correlations were converted into Fisher’s Z-scores for computational purposes and converted back into correlations for presentation [56]. For ease of interpretation, correlations of |0.10|, |0.30|, and |0.50| are considered small, moderate, and large effect sizes, respectively [57,58]. Variance, standard error, 95% confidence interval, and statistical significance for each effect size were computed. 

When at least three studies [59,60] were available on the same association, a meta-analysis was conducted using the software ProMeta 3.0 to obtain an overall estimate. The random-effect model was used as a conservative approach to account for different sources of variation among studies (i.e., within-study variance and between-studies variance; [61]). Moreover, heterogeneity across studies was assessed with the Q statistic to test if it was statistically significant and the I^2^ to estimate it (with values of 25%, 50%, and 75%, respectively, denoting a low, moderate, and a high proportion of dispersion in the observed effects that would remain should sampling error be removed; [62]). Moderator analyses were used to test which factors can account for heterogeneity [63]. Different moderators were tested using meta-regression (for numerical moderators, such as the age of participants and time-lag between waves) when at least three studies for each moderator level were available [60]. Finally, publication bias was examined through the visualization of the funnel plot (i.e., a scatter plot of the effect sizes estimated from individual studies against a measure of their precision, such as their standard errors). Without bias, the plot would be shaped as a symmetrical inverted funnel. However, since smaller or non-significant studies are less likely to be published, studies in the bottom left-hand corner of the plot are often omitted. To evaluate the funnel plot, we also employed Egger’s regression method [64], which statistically tests the asymmetry of the funnel plot, with non-significant results indicative of the absence of publication bias.

## 3. Results

### 3.1. Study Characteristics

Nineteen studies were included in the systematic review. A summary of the characteristics of the included studies is reported in Table 1. With regards the characteristics of the publication, all the studies were articles published in peer-reviewed journals and in the English language. In terms of year of publication, most of them (68.4%) were published between 2017 and 2021, and the remaining studies were published before 2017 (31.6%). With regards the study design, most of the studies (68.4%) included two-time points, while the remaining included more than three-time points (31.6%). The average time lag between adjacent waves was about one year (M = 12.4 months, SD = 10.3 months), ranging from 1.5 months to 4 years. Only one study [12] used objective measures (i.e., actigraphy) to assess sleep variables, while the remaining relied on subjective measures. Most of the studies (84.2%) reported one or multiple sources of funding. The total number of participants was 21,232 (M = 1516.6, SD = 1410.6). Most samples were gender-balanced (the average percentage of females across samples was 53%; range 39–77%), and the average age at baseline was 14.8 years (SD = 1.9, Range: 11–18.8 years). With regards the context of the studies, except for three samples from Belgium [65], New Zealand, [66], and South Korea [67], most of the studies were conducted in the USA (57.9%) and China (26.3%).

### 3.2. The Interplay between Positive Peer Relationships and Sleep Quality over Time

Regarding the interplay between positive peer relationships and sleep quality, four studies examined this link [67,68,76,81]. Of these, only one [81] evaluated this connection bidirectionally. Therefore, a meta-analytic calculation could be applied to obtain overall estimates only for one direction: the longitudinal association between positive peer relationships at one time point (T1) and sleep quality variables at a later time (T2). Results, summarized in Table 2, showed that the effect was small and did not reach statistical significance (*r* = 0.15, *p* = 0.09). Heterogeneity was high and significant, and results were not affected by publication bias.

### 3.3. The Interplay between Sleep and Negative Peer Relationships over Time

Regarding the interplay between negative peer relationships and sleep quality, 11 studies examined this link. Of these, nine studies evaluated the interplay between negative peer relationships at one time point (T1) and different indicators of sleep quality at a later time (T2). The results of the meta-analytic calculations, summarized in Table 3, showed a significant effect (*r* = −0.22, *p* < 0.001). Heterogeneity was high and significant. However, moderator analyses highlighted that the results were not moderated by the characteristics of the participants (i.e., mean age at T1, *B* = −1.07, *p* = 0.25) or by the characteristics of the design (i.e., time-lag between waves, *B* = −0.25, *p* = 0.64). Furthermore, the visual investigation of the funnel plot suggested a low risk of publication bias that was confirmed by a non-significant Egger’s test.

Moreover, four studies [65,66,69,75] evaluated the longitudinal association between different indicators of sleep quality at one time point (T1) and negative peer relationships at a later time (T2). Therefore, a meta-analytic calculation could be applied, and the results, summarized in Table 3, showed a significant effect (*r* = −0.19, *p* < 0.001). Heterogeneity was moderate and significant. However, the results were not moderated by the characteristics of the participants (i.e., mean age at T1, *B* = 0.68, *p* = 0.54) or of the design (i.e., time-lag between waves, *B* = −0.31, *p* = 0.37). Furthermore, the visual investigation of the funnel plot suggested a low risk of publication bias that was confirmed by a non-significant Egger’s test.

**Table 3:** The interplay between negative peer relationships and sleep quality.

Negative Peer Relationship Variable	Study	Sleep Variable	Main Effect Reported Negative Peer Relationships T1 → Sleep Variables T2	Negative Peer Relationships T1 → Sleep Variables T2 ^a^	Main Effect Reported Sleep Variables T1 → Negative Peer Relationships T2	Sleep Variables T1 → Negative Peer Relationships T2 ^a^	Main Findings
Cyberbullying	Erreygers et al., 2019 [65]	Sleep quality (S)			*r* = −0.20 ***	*r* = −0.20 *** [−0.25, −0.15]	Higher sleep quality is associated with less cyberbullying over time. This association is moderate.
	Liu et al., 2021 [75]	Poor sleep quality (S)	*r* = 0.40 ***	*r* = −0.40 *** [−0.46, −0.34]	*r* = 0.27 ***	*r* = −0.27 *** [−0.34, −0.20]	Poor sleep quality and cyberbullying are reciprocally associated over time. These associations are moderate.
Cybervictimization	Herge et al., 2016 [73]	Sleep disturbances (S)	*r* = 0.20 ***	*r* = −0.20 *** [−0.26, −0.14]			Negative peer relationships are associated with higher sleep disturbances symptoms and excessive sleep duration over time. These associations are moderate.
		Excessive sleep duration (S)	*r* = 0.19 ***	*r* = −0.19 *** [−0.25, −0.13]			
	Jose and Vierling, 2018 [66]	Sleep duration (S)	*r* = −0.04	*r* = −0.04 [−0.09, 0.01]	*r* = −0.09 ***	*r* = −0.09 *** [−0.14, −0.04]	Cybervictimization experiences are reciprocally associated with shorter sleep duration over time. These associations are moderate.
Peer Victimization	Chang et al., 2017 [72]	Sleep disturbances (S)	*r* = 0.15 ***	*r* = −0.15 *** [−0.19, −0.11]			Peer victimization is associated with higher sleep disturbances over time. The association is moderate.
	Chang et al., 2019 [70]	Sleep disturbances (S)	*r* = 0.06 ** (males) *r* = 0.18 *** (females)	*r* = −0.06 ** [−0.10, −0.02] (males) *r* = −0.18 ** [−0.22, −0.14] (females)			Peer victimization is associated with higher sleep disturbances over time. The association is small.
	Chang et al., 2018 [71]	Sleep disturbances (S)	*r* = 0.25 ***	*r* = −0.25 *** [−0.28, −0.22]			Negative peer relationships are associated with higher levels of sleep disturbances over time. This association is moderate.
	Lepore and Kliewer, 2013 [74]	Sleep disturbances (S)	*r* = 0.28 ***	*r* = −0.28 *** [−0.37, −0.19]			Peer victimization experiences are associated with higher levels of sleep problems over time. These associations are moderate.
	Herge et al., 2016 [73]	Sleep disturbances (S)	(relational) *r* = 0.09 ** (reputational) *r* = 0.20 *** (overt) *r* = 0.11 **	(relational) *r* = −0.09 ** [−0.15, −0.03] (reputational) *r* = −0.20 *** [−0.26, −0.14] (overt) *r* = −0.11 ** [−0.17, −0.05]			Different dimensions of peer victimization (i.e., relational, reputational, overt) are associated with higher sleep disturbances symptoms and excessive sleep duration over time. These associations are small.
		Excessive sleep duration (S)	(relational) *r* = 0.14 *** (reputational) *r* = 0.17 *** (overt) *r* = 0.09 **	(relational) *r* = −0.14 *** [−0.20, −0.08] (reputational) *r* = −0.17 *** [−0.23, −0.11] (overt) *r* = −0.09 ** [−0.15, −0.03]			
	Tu et al., 2019 [80]	Sleep quality (S)	*r* = −0.46 ***	*r* = −0.46 *** [−0.62, −0.30]			Peer victimization is negatively associated with sleep quality over time. This association is moderate.
Social problems with peers	Brodar et al., 2020 [69]	Sleep disturbances (S)	*r* = 0.20 ***	*r* = −0.20 *** [−0.28, −0.12]	*r* = 0.22 ***	*r* = −0.22 *** [−0.30, −0.14]	Sleep disturbances are reciprocally associated with negative peer experiences over time. These associations are moderate.
	Roberts et al., 2008 [77]	Sleep disturbances (S)	OR = 1.59 * (1.10–2.30)				Sleep disturbances were associated with higher levels of negative peer relationships.
Overall effect	k		ES [95% CI]	Q	I^2^		Eggers’ test
Indicators of Negative peer relationships T1 → Indicators of Sleep Quality T2	9		−0.22 *** [−0.28, −0.15]	117.05 ***	93.17		−4.13
Indicators of Sleep Quality T1 → Indicators of Negative Peer Relationships T2	4		−0.19 *** [−0.27, −0.11]	22.01 ***	86.37		−6.38

Note: S = subjective sleep assessment; *r* = Pearson’s correlation coefficient and confidence intervals in square brackets; OR = odds ratio and confidence intervals in parenthesis; k = number of studies; ES = effect size; Q = heterogeneity test; I^2^ = heterogeneity estimate. *** *p* < 0.001, ** *p* < 0.01, * *p* < 0.05. ^a^ = Effect sizes expressed as Pearson’s correlations. To compute the overall meta-analytic summary, the effect sizes of studies were recoded so that peer negative relationships at T1 were related to higher sleep quality parameters at T2, and higher sleep quality at T1 was related to more peer negative relationships at T2. Since Roberts et al. [78] only reported computed odds ratio and confidence interval, it was not possible to include it in the meta-analytic calculations.

### 3.4. The Interplay between Emotional and Behavioral Issues Related to Peer Relationships and Sleep over Time

Four studies [12,78,79,82] evaluated the reciprocal longitudinal associations between sleep quality and emotional (i.e., loneliness, fear of missing out) and behavioral (i.e., risky sexual behavior, interpersonal functioning) issues of peer relationships over time. The main findings are summarized in Table 4. 

As far as emotional issues are concerned, on the one hand, one study (the only one that measured sleep quality through actigraphy) found no significant bidirectional relationship between sleep quality and loneliness [12]. On the other hand, one study [82] found a bidirectional relationship between bedtime procrastination and fear of missing out and a unidirectional association between short sleep duration and higher fear of missing out. Regarding behavioral issues, one study examined the impact of sleep quality and duration on the likelihood of implementing risky sexual behaviors [79]. Results showed that this risk was associated with shorter sleep duration but not poor sleep quality. Finally, one study [78] found no significant association between short sleep duration and interpersonal functioning over time.

## 4. Discussion

Sleep quality represents a complex process determined by a wide range of biological, behavioral, social, and cultural factors, which must thus be considered by adopting a developmental ecological systems perspective [41]. Principally, sleep quality is a crucial part of adolescents’ well-being since they experience many changes in their life schedules and social contexts [38]. One of the most important contexts in which adolescents are embedded is the peer social context, wherein young people can experience different kinds of contact, both positively and negatively connotated, which might impact their social experience and, in turn, their well-being [5]. 

Despite an increasing interest in evaluating the associations between peer relationships and sleep quality, a comprehensive understanding of their reciprocal links is lacking. The current study addressed this gap by systematically reviewing longitudinal research focusing on the interplay between adolescents’ sleep quality and peer relationships (i.e., positive, negative, emotional, and behavioral issues related to peer relationships). Overall, findings from this review showed that (a) positive peer relationships and sleep quality were not associated over time; (b) negative peer relationships and sleep quality were bidirectionally associated over time; (c) few studies evaluated the bidirectional relations between emotional and behavioral issues and sleep quality, showing links with sleep schedule and duration, but not with sleep quality.

### 4.1. Count Your Blessings: The Reciprocal Associations between Positive Peer Relationships and Sleep Quality 

First, this systematic review examined the associations between positive peer relationships and the sleep quality of adolescents. Available longitudinal studies mainly tackled the association between different kinds of interaction with peers and sleep quality at a later time point. The overall effect size was small and did not reach statistical significance. Nevertheless, it suggests that positive peer relationships may enhance adolescents’ health over time. Considering that the average time-lag was about one year and a half [67,68,76,81] and that often longitudinal effect sizes can be reduced in magnitude because of the control for past levels on the outcome (i.e., stability effects), this result is still worthy of attention [83]. Furthermore, a closer look at the data indicated that the longitudinal associations were generally more robust when the focus was on general peer relationships [67,76,81] than when the attention was limited to romantic relationships [68]. This heterogeneity suggests the importance of evaluating different aspects and types of positive peer relationships, considering the role that diverse bonds might play in affecting adolescents’ well-being [84,85].

In contrast, due to the exiguous number of studies examining the impact of sleep quality on subsequential positive peer relationships, it was not possible to obtain an overall estimate for the effect size in this direction. The only available study on this highlighted that sleep disturbances had detrimental effects on later relationships with peers [81]. Since sleep quality could play a crucial role in impacting individuals’ mood [47], higher sleep quality may be associated with more positive peer relationships because it could facilitate adolescents’ behavioral and emotional regulation, which would, in turn, lead to more positive interactions with peers and friends [81]. Given the role of sleep quality in explaining differences in the quality of interpersonal relationships, it is of utmost importance to tackle this aspect further in future research. 

### 4.2. Engaged in a Vicious Circle: The Reciprocal Associations between Negative Peer Relationships and Sleep Quality 

Second, the current review investigated the longitudinal interplay between negative peer experiences (i.e., peer victimization, cyberbullying, cybervictimization, and social problems with peers) and the sleep quality of adolescents. The majority of available studies examined to what extent experiencing negative peer relationships might affect subsequent sleep quality. The overall effect size was small-to-moderate and significant, highlighting the detrimental consequences of negative peer relationships on sleep quality. Specifically, all the dimensions taken into account showed a small-to-moderate association with poor sleep quality [69,77], sleep duration [66,73], and sleep disturbances [70,71,72,73,74,80]. This effect is in line with the previous literature highlighting the impact of negative social experiences with peers on adolescents’ sleep concurrently and over time [26]. 

Additionally, studies included in the review also examined whether sleep quality affects negative peer relationships over time. The overall effect size was small-to-moderate and significant. In this regard, results indicated that good sleep quality could be a protective factor against cyberbullying [65]. In contrast, poor sleep quality could have a detrimental effect on the quality of peer relationships [75]. Specifically, the review results showed that sleep disturbances are associated with negative online peer experiences (i.e., cyberbullying, cybervictimization). This evidence adds to the literature on the negative consequences of online experiences (i.e., problematic social network use) on adolescents’ functioning [86,87]. These results, in conjunction with the ones discussed above, are worthy of attention as they underscore that the relations between poor sleep quality and negative peer experiences are consistent and reciprocal. 

### 4.3. A Glance under the Surface: The Reciprocal Association between Emotional and Behavioral Issues Related to Peer Relationships and Sleep over Time

Finally, the current systematic review considered additional dimensions that are particularly relevant for understanding the quality of peer relationships. Due to the exiguous number of studies examining these factors, it was not possible to conduct a meta-analytical test on these links. While interpersonal functioning (e.g., [78]) was not related to sleep quality dimensions, fear of missing out and engagement in risky sexual behaviors were associated with adolescents’ sleep duration and schedule [79,82]. These factors were mainly associated with bedtime procrastination, underscoring, in line with the literature, the critical role of the social system changes on sleep health and practices [88].

Only one study [12] tested the longitudinal association between loneliness and sleep quality, reporting no significant findings. Nevertheless, it should be noted that among the available studies, this is the only one that used objective measures to assess sleep quality, in contrast with other studies which used subjective measures. While subjective and objective measurements are firmly related, some differences related to subjective biases and overestimating sleep duration might occur [89]. Moreover, previous studies assessing the relationship between loneliness and well-being considered different age groups, such as adults [43], and the elderly (for a review, [90]), suggesting that there may be a cohort difference in these associations.

### 4.4. Limitations of the Literature and Future Research Directions

The current review highlighted several limitations of the literature on the interplay of peer relationships and sleep quality in adolescence. First, most of the available studies considered multiple dimensions separately but a comprehensive understanding of the relations between multiple facets of peer relationships and multiple indicators of sleep quality over time is lacking. Therefore, it would be essential to disentangle how different peers (e.g., romantic partners or friends; [91]) and specific components of the relationships (e.g., disclosure, support; [92]) affect adolescents’ sleep quality. 

Second, although all the studies included in the review adopted a longitudinal design, only a few of them addressed the longitudinal interplay between sleep quality and peer relationships considering effects in both directions (i.e., from peer relationships to sleep quality, and vice versa, from sleep quality to peer relationships). Future research could benefit from uncovering the bidirectional interplay between peer relationships and sleep quality. In doing so, it is crucial to design multi-wave studies that distinguish which effect unfolds in the short, medium, or long term. 

Third, another limitation that emerged from the current review is the lack of research adopting a combination of sleep measures. Almost all studies used subjective methods to assess sleep duration, quality, and disturbances, while only one used objective measures (i.e., actigraphy; [12]). If, on the one hand, self-reported sleep information may produce inaccurate estimates due to multiple biases (e.g., social desirability and reporting of time in bed rather than sleep duration), on the other hand, objective measures do not consider the subjective perception of sleep quality [89]. Therefore, future research could benefit from a combination of both assessment methods to provide a reliable and comprehensive picture of the antecedents and consequences of sleep quality [93]. 

Fourth, this systematic review indicated that the associations between peer relationships and sleep quality range from small-to-moderate. Future research is needed to understand the underlying mechanisms. For instance, self-concept has been associated with health-related behaviors and emotional self-regulation, which can improve sleep [3]. Moreover, previous research has highlighted the impact of ethnic discrimination on adolescents’ sleep [94]. On the other side, adolescents’ sleep disturbances may exacerbate emotional and behavioral dysregulation [95,96], which in turn has detrimental effects not only on their mental health [97] but can also lead to aggressive behaviors against peers [98]. Therefore, multiple mediators (e.g., self-concept, ethnic identity and discrimination, mental health conditions, and emotional and behavioral dysregulation) could be considered in future studies as critical factors to explain the interplay between peer relationships and sleep quality.

Finally, more research is needed to identify potential moderating factors that could either amplify or weaken the association between peer relationships and sleep quality. In this regard, in line with the developmental-ecological [4,99] and the transactional [100] models, the experience that adolescents have in other ecological contexts, such as those of the family and school, can enhance or reduce the effect of peer relationship dimensions on sleep quality over time. For instance, parental acceptance, which can promote adolescents’ emotional security in their relationships, can positively interact with peer relationships, amplifying their protective role for sleep quality [101]. Thus, future research could benefit from examining how the different social experiences of adolescents can interact in explaining their sleep quality. 

### 4.5. Theoretical and Practical Implications

This review has crucial theoretical and practical implications. From a theoretical perspective, it advanced the understanding of adolescents’ psycho-social development by highlighting the connection between, on the one hand, good practice and experiences with positive outcomes and, on the other hand, bad experiences with detrimental effects on adolescents’ functioning (“Good goes together with good, and bad with bad, in adolescent development”; [102]). Moreover, the current research results add to the literature on the erosion effect of adolescents’ maladjustment on the quality of their relationships [102] by highlighting the importance of considering sleep quality as a key factor of well-being. 

From a practical perspective, this knowledge can help to design evidence-based preventive and promotion interventions. On the one hand, the findings of this review indicate that interventions finalized to improve adolescents’ social relationships with their peers (e.g., intervention to promote resilience against online peer victimization; [103]) might have positive implications for their health and well-being, also improving their sleep quality. On the other hand, interventions focused on improving adolescents’ sleep quality (e.g., educational sleep hygiene programs; [104]), may also enhance the quality of their social experiences across multiple contexts, including peer relationships. 

## 5. Conclusions

The current systematic review with meta-analysis provides an overview of the state-of-the-art of longitudinal associations between peer relationships and the sleep quality of adolescents, offering a nuanced synthesis of the literature on this topic. Overall, the results highlighted a bidirectional relation between peer relationships and sleep quality, underlining the importance of the peer social context in influencing adolescents’ health and well-being. Notably, on the one hand, positive peer relationships were considered potential protective factors against sleep disturbances. On the other hand, results suggested the existence of a vicious circle between negative peer relationships and sleep health. Specifically, negative peer experiences in both real (i.e., peer victimization, social problems with peers) and online (i.e., cyberbullying, cybervictimization) contexts are associated with poor sleep quality and, conversely, poor sleep quality may have detrimental consequences on peer relationships. In addition, other emotional and behavioral features of peer relationships were considered, suggesting a meaningful association between sleep quality and fear of missing out [82]. Finally, this systematic review offers an agenda for future research and highlights practical implications for interventions aimed at enhancing adolescents’ positive development. 

## Figures and Tables

**Table 1:** Characteristics of studies included in the systematic review.

Study	Characteristics of Studies				Characteristics of the Participants				
Authors and Year	Funding	Waves	Time Lag	N Baseline	N Follow-Up	% Girls	Mean Age T1	Country	% Ethnicity
Aalsma et al., 2012 [68]	Yes	2	1 year	748	160	50	15.60	USA	White 89%; Black 8%
Brodar et al., 2020 [69]	Yes	2	7 months	522	502	58	14.22	USA	Hispanic/Latin 91.2%
Chang et al., 2019 [70]	Yes	2	2 years	n.a.	4072	50	14.67	China	n.a.
Chang et al., 2018 [71]	Yes	2	2 years	2854	2854	49	16	Taiwan	n.a.
Chang et al., 2017 [72]	Yes	4	1 year	2006	2006	50.10	14.68	Taiwan	n.a.
Erreygers et al., 2019 [65]	Yes	2	4 months	1746	1590	55	13.50	Belgium	n.a.
Herge et al., 2016 [73]	Yes	3	1.5 months	1177	1162	52	15.80	USA	Hispanic 80%; White 84%, Black 12%, Asian 4%
Jose and Vierling, 2018 [66]	Yes	3	1 year	2179	1774	n.a.	n.a.	New Zealand	New Zealand European 59%; Māori 28%, Other 15% (chiefly Pacifica and Asian New Zealanders)
Lepore and Kliewer, 2013 [74]	Yes	2	6 months	n.a.	498	56	14.45	USA	White/Caucasian 43%; Latino/a 24%; Black/African American 24%; other race/ethnicity 9%.
Liu et al., 2021 [75]	No	2	8 months	879	671	39	14.02	China	n.a.
Maume et al., 2017 [76]	Yes	2	4 years	n.a.	974	50	n.a.	USA	n.a.
Roberts et al., 2008 [77]	Yes	2	1 year	4175	3134	49	n.a.	USA	European 37.0%, African 34.6%, Latino 23.7%, and other American 4.7%
Roberts et al., 2009 [78]	No	2	1 year	4175	3134	49	n.a.	USA	European 37.0%, African 34.6%, Latino 23.7%, and other American 4.7%
Sladek and Doane, 2015 [12]	Yes	3	6 months	82	71	77	18.85	USA	Caucasian 52%, Latino/Hispanic descent 25%, African American 6%, Asian American/Pacific Islander 4%, and multiracial 13%.
Troxel et al., 2019 [79]	Yes	4	1 year	n.a.	1850	57	16.23	USA	White 21.6%, Hispanic 44%, Asian 21.1%, Black 2.5%, Other race 10.9%
Tu et al., 2019 [80]	Yes	2	10 months	123	99	52	12.78	USA	European Americans 58.5%, African Americans 35%, other races/ethnicities 6.5%
Tu and Cai, 2020 [81]	No	2	7.4 months	100	89	49	11.05	USA	European Americans 57–63%; African Americans 8–11%; Hispanic/Latino 14–17%; Asians 6–9%; other race/ethnicity 2–12% (e.g., biracial).
Yoo, 2020 [67]	Yes	4	1 year	n.a.	4335	48.50	12.95	South Korea	n.a.
Zhang et al., 2021 [82]	Yes	2	6 months	466	318	64	16.92	China	n.a.

Note. N.a. = not applicable.

**Table 2:** The interplay between positive peer relationships and sleep quality.

Peer Relationship Variable	Study	Sleep Variable	Main Effect Reported Positive Peer Relationships T1 → Sleep Variables T2	Positive Peer Relationships T1 → Sleep Variables T2	Main Effect Reported Sleep Variables T1 → Positive Peer Relationships T2	Sleep Variables T1 → Positive Peer Relationships T2	Main Findings
Romantic relationships	Aalsma et al., 2012 [68]	Sleep duration (S)	*β =* −0.02 (−0.16, 0.12)	*r* = −0.02 [−0.18, 0.14]			Partner behavior in romantic relationships is not associated with sleep duration over time.
Relationships with peers	Maume et al., 2017 [76]	Sleep duration (S)	*β* = 0.07	*r* = 0.12 *** [0.06, 0.18]			Positive peer relationships are associated with longer sleep duration over time. The effect is small.
	Tu and Cai, 2020 [81]	Sleep disturbances (S)	*r* = −0.38 ***	*r* = 0.38 *** [0.20, 0.56]	*r* = −0.30 **	*r* = 0.30 ** [0.11, 0.49]	Positive peer relationships and lower levels of sleep disturbances are reciprocally associated over time. These effects are moderate.
	Yoo, 2020 [67]	Sleep duration (S)	*β =* −0.05 SE: 0.01 (cohort 1); *β =* 0.02 SE: 0.01 (cohort 2)				Positive peer relationships are not associated with sleep duration in the first and second cohorts.
Overall effect	k	ES [95% CI]	Q	I^2^	Eggers’ test		
Different Indicators of Positive Peer Relationships T1 → Different Indicators of Sleep Quality T2	3	0.15 [−0.03, 0.32]	9.80 **	79.60	1.11		

Note. *β* = Standardized regression coefficient and standard error estimate or confidence interval in parenthesis; *r* = Pearson’s correlation coefficient (confidence intervals are reported between square brackets). k = number of studies; ES = effect size; Q = heterogeneity test; I^2^ = heterogeneity estimate. *** *p* <0.001, ** *p* < 0.01; S = subjective sleep assessment. ^a^ = Effect sizes expressed as Pearson’s correlations. To compute the overall meta-analytic summary, the effect sizes of studies were recoded so that positive peer relationships were related to higher sleep quality at T2. Since [67] only reported the effect of change in positive peer relationships on sleep duration at T2, it was not possible to include it in the meta-analytic calculations.

**Table 4:** The interplay between emotional and behavioral issues related to peer relationships and sleep over time.

	Study	Sleep Variable	Main Effect Reported Emotional and Behavioral Issues T1 → Sleep Variables T2	Main Effect Reported Sleep Variables T1 → Emotional and Behavioral Issues T2	Main Findings
Interpersonal functioning	Roberts et al., 2009 [78]	Short sleep duration (S)		OR = 1.19 [0.73; 1.94]	Sleep duration is not associated with interpersonal functioning over time.
Loneliness	Sladek and Doane, 2015 [12]	Sleep duration (O)	*r =* −0.01	*r =* −0.07	There is no association between sleep quality indicators and loneliness over time.
		Sleep latency (O)	*r =* −0.02	*r =* −0.01	
Risky sexual behaviour	Troxel et al., 2019 [79]	Sleep duration (S)		OR Short vs. sufficient sleepers/Weekday = 1.14 [0.91, 1.42] OR Short vs. intermediate/Weekend = 1.02 [0.82, 1.28]	Shorter weekend sleep duration is associated with higher levels of risky sexual behavior over time.
		Sleep variability (S)		OR Sleep variability low vs. high = 1.97 [1.19, 3.26]	Shorter weekday/weekend variability in sleep duration is associated with higher levels of risky sexual behavior over time.
		Sleep quality (S)		OR Poor vs. High Sleep Quality = 0.93 [0.72, 1.21]	Sleep quality is not associated with risky sexual behavior over time.
Fear of missing out	Zhang et al., 2021 [82]	Sleep duration (S)	*r* = −0.10	*r* = −0.20 ***	Sleep duration is negatively associated with fear of missing out over time; the association is moderate.
		Bedtime procrastination (S)	*r* = 0.19 **	*r* = 0.20 ***	Bedtime procrastination and fear of missing out are reciprocally associated over time. These associations are moderate.

Note: S = subjective sleep assessment; O = objective sleep assessment; T = time; OR = odds ratio and confidence interval in parenthesis; *r* = Pearson’s correlation coefficient; *** *p* < 0.001, ** *p* < 0.01.

## Data Availability

Data from previously published studies were retrieved and analyzed.

## Supplementary Materials

The following supporting information can be downloaded at: https://www.mdpi.com/article/10.3390/ijerph20032017/s1, Document S1: PRISMA Checklist; Document S2: Full query strings used in each database; Document S3: List of screened journals; Document S4: List of most relevant systematic reviews and meta-analysis. References [53,54,105,106,107,108,109,110,111,112,113,114] are cited in the supplementary materials.
